# Avian Influenza A (H5) in Wastewater, July 2024 to February 2025

**DOI:** 10.1001/jamanetworkopen.2025.17286

**Published:** 2025-06-26

**Authors:** Melissa Sutton, Rebecca Falender, Ryan Scholz, David Mickle, Paul Cieslak, Gauri Phatak, Tyler Radniecki

**Affiliations:** 1 Public Health Division, Oregon Health Authority, Portland; 2 School of Chemical, Biological, and Environmental Engineering, Oregon State University, Corvallis; 3 Oregon Department of Agriculture, Salem

## Abstract

This cross-sectional study evaluates levels of influenza A (H5) in wastewater in Oregon and associations with highly pathogenic avian influenza livestock outbreaks and bulk tank milk surveillance.

## Introduction

Systematic testing of wastewater can detect and monitor emerging pathogens, including highly pathogenic avian influenza (HPAI) A (H5) during the current outbreak in the US.^1^ Interpretation of influenza A (H5) detections in wastewater is complex because current methods do not distinguish between human and animal contributors.^2^ Influenza A (H5) has been detected in wastewater adjacent to dairy cattle outbreaks, likely due to dairy processing discharge.^3^ Poultry outbreaks and wild birds may also contribute to influenza A (H5) in wastewater.^1^

In Oregon, influenza A (H5) was detected sporadically in wastewater from September 2021 to July 2024, and no association was observed with livestock outbreaks or dairy processing facilities.^2^ Wild birds are suspected to be the primary contributor of the virus to Oregon wastewater through stormwater and leaking pipes.^2^ In this repeated cross-sectional study, we tested wastewater for the influenza A (H5) subtype from July 8, 2024, to February 13, 2025, and investigated associations with HPAI livestock outbreaks and bulk tank milk (BTM) surveillance.

## Methods

Wastewater samples collected from communities participating in statewide wastewater surveillance were tested for the influenza A (H5) subtype from July 8, 2024, to February 13, 2025. Twenty-four–hour composite samples were collected from wastewater treatment facilities 1 to 2 times weekly, filtered, stabilized, extracted, and analyzed for the influenza A (H5) subtype by digital reverse transcription polymerase chain reaction, as previously described.^2^ Wastewater viral concentrations were quantified as log_10_ gene copies normalized for flow and population. A piecewise linear regression model was used to evaluate wastewater H5 percent positivity for a breakpoint. A Wald test was used to test for a difference in the slope of the segmented relationship; *P* < .05 was considered significant. A 2-sided Fisher exact test was used to examine the association between detections in wastewater and animal outbreaks within the sewershed within the preceding 30 days and BTM detections. BTM surveillance in Oregon is conducted in accordance with the US Department of Agriculture national milk testing strategy.^4^

This study followed the Strengthening the Reporting of Observational Studies in Epidemiology (STROBE) reporting guideline. This activity was reviewed by the Oregon Health Authority, deemed not research, and conducted consistent with federal law. Data were analyzed using RStudio, version 4.4.1 (R Project for Statistical Computing).

## Results

**Figure 1.**
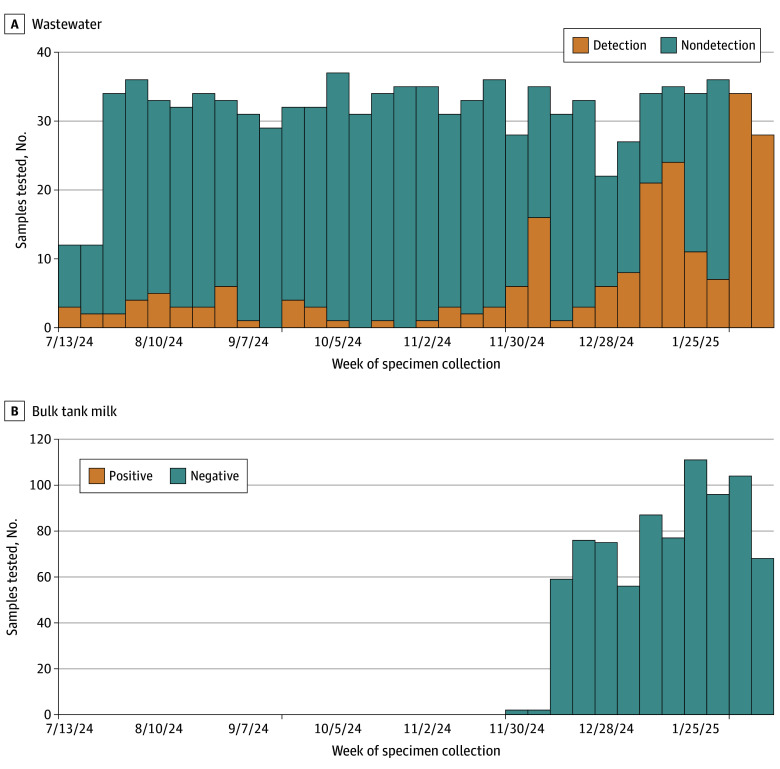
Number of Samples Tested for Influenza A (H5) in Wastewater and Bulk Tank Milk by Epidemiologic Week in Oregon, July 8, 2024 to February 13, 2025

**Figure 2.**
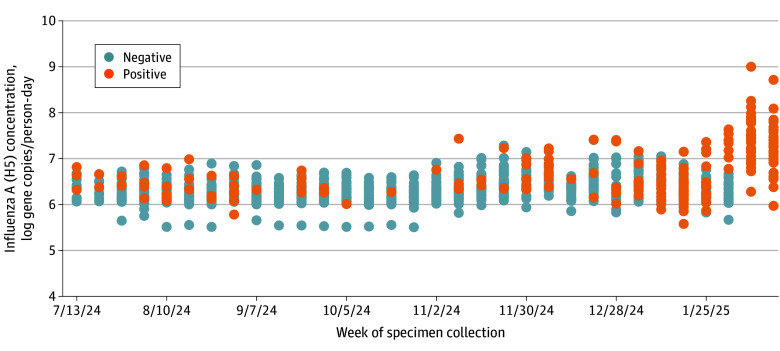
Concentration of Influenza A (H5) in Wastewater by Epidemiologic Week in Oregon, July 8, 2024 to February 13, 2025 Each dot represents a unique sample tested.

From July 8, 2024, to February 13, 2025, 999 wastewater samples were collected from 38 communities; 212 (21.2%) tested positive for the influenza A (H5) subtype (Figure 1A). Weekly mean (SD) and median (IQR) positivity were 21.5% (26.5%) and 10.4% (5.2%-25.6%); weekly positivity (piecewise linear regression model breakpoint estimate, 23.7 weeks; 95% CI, 20.5-26.8 with a significant difference in slope; *P* < .001) and virus levels increased after December 14, 2024, reaching 100% by the end of the study period (Figure 2).

There was 1 HPAI poultry outbreak within a sewershed participating in surveillance within 30 days. Oregon has not had any dairy cattle outbreaks. No association with animal outbreaks was identified. BTM surveillance began November 25, 2024; all 813 BTM samples tested negative for influenza A (H5) (Figure 1B). During the surveillance period, 1745 influenza A-positive specimens were subtyped at the Oregon State Public Health Laboratory and 1 Oregon case of influenza A (H5) was detected.

## Discussion

In this study, the influenza A (H5) subtype was detected in 21.2% of wastewater samples in Oregon. Weekly percentage positivity increased in mid-December 2024, reaching 100% by the end of the study period. There was no association with HPAI poultry outbreaks, and no virus was detected through BTM surveillance.

Historically, influenza A (H5) detections in Oregon wastewater have not been associated with livestock outbreaks or dairy processing facilities and are suspected to stem from wild bird inputs. This study reinforces those findings while offering additional evidence through BTM surveillance. The increase in weekly positivity during the study period coincides with emergence of the D1.1 genotype in wild birds and the overwintering period for many Oregon waterfowl.^5,6^ One limitation of this study is that current surveillance testing methods do not distinguish the pathogenicity of avian influenza in wastewater. Our results highlight the importance of a One Health approach to the interpretation of wastewater surveillance for this emerging pathogen.

## Notes

Supplement.
Data Sharing Statement
