# Supplementary material for: Avian Influenza A (H5) in Wastewater, July 2024 to February 2025
Source: JAMA Netw Open. 2025 Jun 26;8(6):e2517286. doi: 10.1001/jamanetworkopen.2025.17286 (PMC12551649; doi:10.1001/jamanetworkopen.2025.17286)
Supplement: Supplement. — Data Sharing Statement [file jamanetwopen-e2517286-s001.pdf]

Data Sharing Statement

Sutton. Avian Influenza A (H5) in Wastewater, July 2024 to February 2025. *JAMA Netw Open*. Published June 26, 2025. doi:10.1001/jamanetworkopen.2025.17286

Data
Data available: Yes
Data types: Data (not involving human participants)
How to access data: https://www.cdc.gov/nwss/rv/wwd-h5.html
When available: beginning date: 03-07-2025

Supporting Documents
Document types: None

Additional Information
Who can access the data: Open access
Types of analyses: Open access
Mechanisms of data availability: Open access
Any additional restrictions: N/A
